# Genomic prediction using subsampling

**DOI:** 10.1186/s12859-017-1582-3

**Published:** 2017-03-24

**Authors:** Alencar Xavier, Shizhong Xu, William Muir, Katy Martin Rainey

**Affiliations:** 1 Department of Agronomy, Purdue University, 915 W. State St., Lilly Hall, West Lafayette, IN 47907 USA; 2 Department of Plant Science, University of California, 3134 Batchelor Hall, Riverside, CA 92521 USA; 3 Department of Animal Science, Purdue University, 915 W. State St., Lilly Hall, West Lafayette, IN 47907 USA

**Keywords:** Genome-wide selection, Bayesian analysis, Bootstrapping

## Abstract

**Background:**

Genome-wide assisted selection is a critical tool for the genetic improvement of plants and animals. Whole-genome regression models in Bayesian framework represent the main family of prediction methods. Fitting such models with a large number of observations involves a prohibitive computational burden. We propose the use of subsampling bootstrap Markov chain in genomic prediction. Such method consists of fitting whole-genome regression models by subsampling observations in each round of a Markov Chain Monte Carlo. We evaluated the effect of subsampling bootstrap on prediction and computational parameters.

**Results:**

Across datasets, we observed an optimal subsampling proportion of observations around 50% with replacement, and around 33% without replacement. Subsampling provided a substantial decrease in computation time, reducing the time to fit the model by half. On average, losses on predictive properties imposed by subsampling were negligible, usually below 1%. For each dataset, an optimal subsampling point that improves prediction properties was observed, but the improvements were also negligible.

**Conclusion:**

Combining subsampling with Gibbs sampling is an interesting ensemble algorithm. The investigation indicates that the subsampling bootstrap Markov chain algorithm substantially reduces computational burden associated with model fitting, and it may slightly enhance prediction properties.

**Electronic supplementary material:**

The online version of this article (doi:10.1186/s12859-017-1582-3) contains supplementary material, which is available to authorized users.

## Background

The use of genomic tools has become important for the genetic improvement of complex traits in plants and animals through genome-wide prediction (GWP). GWP provides an interesting solution for the selection of traits with low heritability, such as grain yield in crops and milk production in dairy cattle, as well as for traits that present challenging or expensive phenotyping.

Over the past decade, researchers have tried to overcome the pitfalls of increased computational burden associated with gains in prediction accuracy from GWP of complex traits. Increases in predictive ability (and computational burden) are often associated with better statistical learning properties, such as regularization and variable selection [1]. Hence models with an improved ability to identify patterns provide more robust predictions, but computational costs are involved.

In statistical learning, resampling techniques are common approaches used to turn weak learners into strong learners [2]. Gianola et al. [3] showed that bootstrapping aggregation could improve prediction accuracy of kernel-based genomic best linear unbiased prediction (GBLUP) model in genomic prediction of plant and animals. We hypothesized that a similar approach could apply to whole-genome regression methods, often referred to as the Bayesian alphabet [4].

Besides computational advantages offered by some resampling methods, these techniques may also help to overcome theoretical shortcomings of some of these Bayesian methods, such as the bias of BayesA [5]. The objective of this study was to evaluate the predictive and computational outcomes from the application of a resampling technique ensemble with the Gibbs sampler to a Bayesian ridge regression model.

### Sampling procedures

In addition to the increasing number of markers available over time due to higher density single nucleotide polymorphism (SNP) arrays and even resequencing, computation challenges include the large number of samples from which those genotypes are taken [6]. The computational burden associated with large population sizes is more evident in plant breeding, where hundreds of crosses with large offspring are genotyped and selected every season using GWP. Sampling methods are often necessary to enable such complex statistical procedures in large datasets. Among those, two main classes of sampling techniques are Markov chain Monte Carlo (MCMC) and Bootstrapping.

The MCMC method is possibly the most popular Monte Carlo algorithm with application to linear models, providing a feasible framework to resolve high-dimensional problems (i.e., more parameters than observations) with moderate computer power [7]. Likewise, bootstrapping also provides an interesting framework for solving large-scale problems [8, 9], particularly a method known as subsampling [10] used to reduce data dimensionality.

### Gibbs sampling

Gibbs sampling is a widely used MCMC technique, applied in conjunction with Bayesian methods to generate the posterior distribution of the parameters. The posterior distribution is denoted as $p\left(\varTheta \Big| X\right)$, where $\varTheta$ represents the set of unknown parameters $\varTheta =\left\{{\theta}_1,{\theta}_2,\dots, {\theta}_r\right\}$, and $X$ represents the data. The Gaussian model described in the following section, unknown parameters include the intercept ($\upmu$), the vector of regression coefficients ($\mathbf{b}$) and variance components, as $\varTheta =\left\{\upmu, \mathbf{b},{\upsigma}_{\mathrm{b}}^2,{\upsigma}_{\mathrm{e}}^2\right\}$, whereas the observed data comprises the genotypic information ($\mathbf{X}$) of individuals and phenotype ($\mathbf{y}$), as $X=\left\{\mathbf{X},\mathbf{y}\right\}.$

Gibbs sampling algorithms are based on updating each parameter with samples drawn from the full-conditional posterior distribution, one parameter at a time while holding every other parameter constant. Each parameter $\theta$ is sampled from

1 $$ p\left(\theta \left| X\right.\right)\propto f\left( X\left|\theta \right.\right)\pi \left(\theta \right),\forall \theta \in \varTheta, $$

where $p\left(\theta | X\right)$ denotes the posterior distribution of $\theta$, the likelihood is expressed as $f\left( X\Big|\theta \right)$ and the prior distribution of $\theta$ is $\pi \left(\theta \right)$.

In most implementations, regression coefficients are sampled individually from normal distributions whereas variance components are sampled from scaled inverse chi-squared distributions [4, 5]. Every time a parameter (i.e., regression coefficients and variance components) or a conjugated prior is updated, its value is stored as samples of the posterior distribution. The final Bayesian estimator is the expectation of the posterior distribution, obtained as the mean of the posterior distribution.

### Bootstrapping aggregation

A natural strategy to increase prediction accuracy is to build and combine multiple prediction models generated from samples of a large dataset, averaging the outcome predictor [11]. Bootstrapping aggregation, or simply ‘bagging’, is implemented in linear models by fitting the function ${\widehat{f}}_1\left(\mathrm{x}\right),{\widehat{f}}_2(x),\dots, {\widehat{f}}_B(x)$ with $B$ bootstrapped samples of the dataset and the final model, with reduced variance, will be given by

2 $$ {\widehat{f}}_{avg}(x)=\frac{1}{B}{\displaystyle {\sum}_{b=1}^B{\widehat{f}}_b(x),}\kern0.14em  x\subset X. $$

Regression coefficients are stored each time the model is fitted, hence generating an empirical distribution of each parameter. Bagging parameters are obtained as the mean of this distribution.

With bootstrapping, when samples are obtained with replacement, the number of observations sampled is commonly the same size as the initial dataset, recognizing that some observations may be sampled more than once. When bootstrapping is performed with fewer samples than the original number of observations, sampling can proceed either with or without replacement. The latter case is known as subsampling.

### Subsampling bootstrap Markov chains

MCMC and Bootstrap are usually implemented separately, such that some studies have attempted to compare the performance of these samplers [12]. Nevertheless, both methods can be co-implemented. A co-implementation that is becoming popular in the context of big data is a technique known as subsampling bootstrap Markov chain (SBMC). In this algorithm, the Markov chain update mechanism is performed upon a subset ($x$) of the whole data ($X$) and a different subset is used to update the parameters in each round of MCMC. Therefore, each parameter is sampled from the posterior distribution

3 $$ p\left(\theta \left| x\right.\right)\propto f\left( x\left|\theta \right.\right)\pi \left(\theta \right),\forall \theta \in \varTheta, x\subset X. $$

The concept of subsampling Gibbs sampler was first presented by Geyer [13] and some predictive properties were further investigated by MacEachern and Peruggia [14]. Regarding the applications to genome-wide prediction of complex traits, SBMC can be used to update the regression coefficients [15], hence increasing the computational performance of model fitting.

## Methods

### Statistical model

The family of whole-genome regression methods is a standard set of models widely applied for genomic prediction [4]. Among these, Bayesian ridge regression is a regularized model that assigns the same variance to every marker. The linear model is described as follows:

4 $$ \mathbf{y}=1\upmu +\mathbf{X}\mathbf{b}+\mathbf{e} $$

where $\mathbf{y}$ is the response variable (i.e., the phenotypic information), $\upmu$ is a scalar representing the intercept, $\mathbf{X}$ is the genotypic matrix coded as {0,1,2} for {AA, Aa, aa} where rows correspond to the genotypes and columns correspond to the molecular markers, $\mathbf{b}$ is a vector of regression coefficients that represents the additive value of allele substitutions, and $\mathbf{e}$ is the vector of residuals. In this model, both regression coefficients and residuals are assumed to be normally distributed as $\mathbf{b}\sim \mathrm{N}\left(0,\mathrm{I}{\upsigma}_{\mathrm{b}}^2\right)$ and $\mathbf{e}\sim \mathrm{N}\left(0,\mathrm{I}{\upsigma}_{\mathrm{e}}^2\right)$. The variances are assumed to follow a scaled inverse chi-squared distribution with a given prior shape (S) and prior degrees of freedom ($\upnu$), thus ${\upsigma}_{\mathrm{b}}^2\sim {\upchi}^{-2}\left({\mathrm{S}}_{\mathrm{b}},{\upnu}_{\mathrm{b}}\right)$ and ${\upsigma}_{\mathrm{e}}^2\sim {\upchi}^{-2}\left({\mathrm{S}}_{\mathrm{e}},{\upnu}_{\mathrm{e}}\right)$.

High-dimensional methods are regularized to enable fitting the model without losing predictive properties [2]. The regularization of linear models occurs by shrinking regression coefficients, which also biases predictions downwards [1]. The Bayesian ridge regression attempts to estimate regression coefficients with the minimum bias necessary for a satisfying prediction (i.e., minimum variance), a solution referred to as best linear unbiased predictor [4, 5]. As an optimization problem, the loss function to be minimized by the model (equation 4) that balances variance and bias is described as

5 $$ {L}_2={\left(\mathbf{y}-\upmu -\mathbf{Xb}\right)}^{\prime}\left(\mathbf{y}-\upmu -\mathbf{Xb}\right)+\uplambda \left({\mathbf{b}}^{\prime}\mathbf{b}\right) $$

where $\uplambda$ is the regularization parameter, the ratio between the residual variance and the genetic variance of marker effects, as $\uplambda ={\upsigma}_{\mathrm{e}}^2/{\upsigma}_{\mathrm{b}}^2$. For the model in consideration, the regularization parameter assumes a single value for all regression coefficients.

### Coefficient update

Sorensen and Gianola [16] show that the full conditional distribution of regression coefficients for Gibbs sampling from a normal distribution has a closed form. The expectation is derived from the Cholesky decomposition of the left-hand side (LHS) of the mixed model equation. The computational cost of operations such as solving the mixed model equation is described in terms of $n$ observations and $p$ parameters. The cost associated with the Cholesky decomposition is ${p}^3$, making it computationally unfeasible for high-dimensional problems ($p\gg n$), such as whole-genome regression methods. On the other hand, the Gauss-Seidel residual updating (GSRU) algorithm [15] has a computational cost of $3 p n$, which is much lower than for the Cholesky decomposition in this scenario. A Gibbs sampler based on GSRU updates the ${\mathrm{j}}^{\mathrm{th}}$ regression coefficient as

6 $$ {\mathrm{b}}_{\mathrm{j}}^{\mathrm{t}+1}\left|\ast \sim \mathrm{N}\left(\frac{{\mathrm{x}}_{\mathrm{j}}'{\mathrm{e}}^{\mathrm{t}}+{\mathrm{x}}_{\mathrm{j}}'{\mathrm{x}}_{\mathrm{j}}{\mathrm{b}}_{\mathrm{j}}^{\mathrm{t}}}{{\mathrm{x}}_{\mathrm{j}}'{\mathrm{x}}_{\mathrm{j}}+{\uplambda}_{\mathrm{j}}},\frac{\sigma_e^2}{{\mathrm{x}}_{\mathrm{j}}'{\mathrm{x}}_{\mathrm{j}}+\uplambda}\right)\right. $$

where ${\mathrm{x}}_{\mathrm{j}}$ is the vector corresponding to the ${\mathrm{j}}^{\mathrm{th}}$ marker and $*$ represents the data and all parameters other than the one being updated. The coefficient update is followed by update of the vector of residual

7 $$ {\mathbf{e}}^{\mathrm{t}+1}={\mathbf{e}}^{\mathrm{t}}+{\mathbf{x}}_{\mathrm{j}}\left({\mathrm{b}}_{\mathrm{j}}^{\mathrm{t}+1}-{\mathrm{b}}_{\mathrm{j}}^{\mathrm{t}}\right). $$

The greatest advantage of GSRU comes from the low computational cost of updating the right-hand side (RHS) of the mixed model equation [15], solving the linear system one parameter at a time without computing $\mathbf{X}'\mathbf{X}$. Subsequently, variance components are updated as

8 $$ {\sigma}_b^2\Big|\ast \sim \frac{{\mathbf{b}}^{\prime}\mathbf{b}+{\mathrm{S}}_{\mathrm{b}}{v}_{\mathrm{b}}}{\chi_{p+{v}_b}^2}\operatorname{}\mathrm{and}{\sigma}_e^2\Big|\ast \sim \frac{{\mathbf{e}}^{\prime}\mathbf{e}+{\mathrm{S}}_e{v}_e}{\chi_{n+{v}_e}^2}.\operatorname{} $$

where ${\mathrm{S}}_{\mathrm{e}}$, ${\upnu}_{\mathrm{e}}$, ${\mathrm{S}}_{\mathrm{b}}$, and ${\upnu}_{\mathrm{b}}$ correspond to the prior parameters “shape” and “degrees of freedom” of the residual and genetic variance, respectively.

### SBMC extension

We here propose incorporating subsampling into the Gibbs sampler. This variation implies sampling a $\uppsi$ fraction of the data ($\uppsi \in \left[0,1\right]$) to update regression coefficients and residual variance in each round of MCMC.

For a matter of notation, let $\overset{\sim }{\mathbf{X}}$ and $\overset{\sim }{\mathbf{e}}$ represent the bagged subsamples, in other words, a fraction of $\mathbf{X}$ and $\mathbf{e}$ that contains $\uppsi$ percent of observations sampled at random in a given round of MCMC. This modified GSRU would have an expected computational cost of $3 p n\uppsi$.

To accommodate bagged samples, sampling algorithms of regression coefficients and residual variance undergo a slight modification. Regression coefficients are updated or sampled as

9 $$ {b}_j^{t+1}\Big|\ast \sim N\left(\frac{{\overset{\sim }{\mathbf{x}}}_{\mathbf{j}}^{\prime }{\overset{\sim }{\mathbf{e}}}^{\mathrm{t}}+\uppsi {\mathbf{x}}_{\mathrm{j}}^{\prime }{\mathbf{x}}_{\mathrm{j}}{\mathrm{b}}_{\mathrm{j}}^{\mathrm{t}}}{\uppsi {\mathbf{x}}_{\mathrm{j}}^{\prime }{\mathbf{x}}_{\mathrm{j}}+{\uplambda}_{\mathrm{j}}},\frac{\sigma_e^2}{\uppsi {\mathbf{x}}_{\mathrm{j}}^{\prime }{\mathbf{x}}_{\mathrm{j}}+{\uplambda}_{\mathrm{j}}}\right)\operatorname{} $$

with subsequent residual update

10 $$ {\overset{\sim }{\mathbf{e}}}^{t+1}={\overset{\sim }{\mathbf{e}}}^t+{\mathbf{x}}_{\mathrm{j}}\left({b}_j^{t+1}-{b}_j^t\right). $$

The entire ${\mathrm{k}}^{\mathrm{th}}$ round of MCMC is updated using the subsampled dataset ${x}^{\mathrm{k}}=\left\{\overset{\sim }{\mathbf{X}},\overset{\sim }{\mathbf{e}}\right\}$. Since the residual variance is a function of the number of observations, its update is slightly modified from equation 8 as

11 $$ {\sigma}_e^2\Big|\ast \sim \frac{{\overset{\sim }{\mathbf{e}}}^{\prime}\overset{\sim }{\mathbf{e}}+{\mathrm{S}}_e{v}_e}{\upchi_{\uppsi \mathrm{n}+{v}_e}^2}.\operatorname{} $$

The sampling procedure above assumes that the variance associated to markers in the subsamples are approximately the same as in the whole data (${\sigma}_{\overset{\sim }{\mathrm{x}}}^2\approx {\sigma}_{\mathrm{x}}^2$). That is, the marker sum of squares ($\mathbf{x}'\mathbf{x}$) is expected to reduce linearly according to the proportion of bag samples ($\uppsi \mathbf{x}'\mathbf{x}$) to avoid recalculating the sum of squares of bagged markers (${\overset{\sim }{\mathbf{x}}}^{\prime}\overset{\sim }{\mathbf{x}}$) for each round of MCMC. In genetic terms, the subset is assumed to have the same allele frequencies as the whole set.

The SBMC algorithm is implemented in the R package bWGR [17] using the ${\mathrm{R}}^2$ rule proposed by Pérez and de Los Campos [18] to estimate prior shapes using the whole data, based on ${\mathrm{R}}^2=0.5$, with the values of prior degrees of freedom set as ${\upnu}_{\mathrm{e}}=5$ and ${\upnu}_{\mathrm{b}}=5$. In the ${\mathrm{R}}^2$ rule [18], prior shapes are estimated as

12 $$ {\mathrm{S}}_{\mathrm{b}}={R}^2\times {\sigma}_{\mathrm{y}}^2\times \frac{\left({v}_{\mathrm{b}}+2\right)}{{\displaystyle {\sum}_{\mathrm{j}}{\upsigma}_{{\mathrm{x}}_{\mathrm{j}}}^2}} $$

and

13 $$ {S}_e=\left(1-{R}^2\right)\times {\sigma}_y^2\times \left({v}_e+2\right). $$

### Dataset

Three datasets available on R packages [18, 19] were chosen to demonstrate the effect of bagging on genomic prediction, including a wheat panel from the International Maize and Wheat Improvement Center (CIMMYT), as the median of grain yield observed in four environments [20]; a mouse panel designed to study body mass index [21] but using only half the SNP panel obtained by skipping every other marker; a soybean panel with eight bi-parental families with elite parents from the SoyNAM project [19] with phenotypes observed in eighteen environments; and a simulated F_2_ population with 10 chromosomes of 50 cM each, genotyped at density of 1 SNP/cM, and with one QTL every 10 cM placed between markers. Heritability of traits was computed by restricted maximum likelihood (REML) upon the animal model with additive kernel [22]. Markers with minor allele frequency below 0.05 were removed. Datasets are summarized in Table 1.

**Table 1:** Summary of datasets used in this study

Species	Population type	Trait	n	$\mathrm{p}$	${\mathrm{h}}^2$	Source
Mouse	Heterogeneous stock	Body mass index	1814	5173	0.146	Legarra et al. [21]
Soybean	Nested Ass. Panel	Grain yield	1079	4307	0.345	Xavier et al. [19]
Wheat	Diverse panel	Grain yield	599	1209	0.434	Crossa et al. [20]
Simulation	Experimental F2	Simulated	400	500	0.516	Technow [23]

### Prediction metrics

Prediction statistics were obtained with a 10-fold cross validation scheme. We fitted the Bayesian ridge regression model using subsampling from 25 to 100%, by increment of 1%, with and without replacement. We set the algorithm for 4000 MCMC iterations to ensure convergence [16], with 500 of burn-in [18].

To determine the efficacy of subsampling, we evaluated the mean square prediction error (MSPE), prediction bias as the slope of linear regression between predictions and observations (${\upbeta}_{\mathrm{y},\widehat{\mathrm{y}}}$), computation time in minutes, and predictive ability as the Pearson’s correlation between predictions and observations ($\mathrm{C}\mathrm{o}{\mathrm{r}}_{\mathrm{y},\widehat{\mathrm{y}}}$).

## Results

**Fig. 1:**
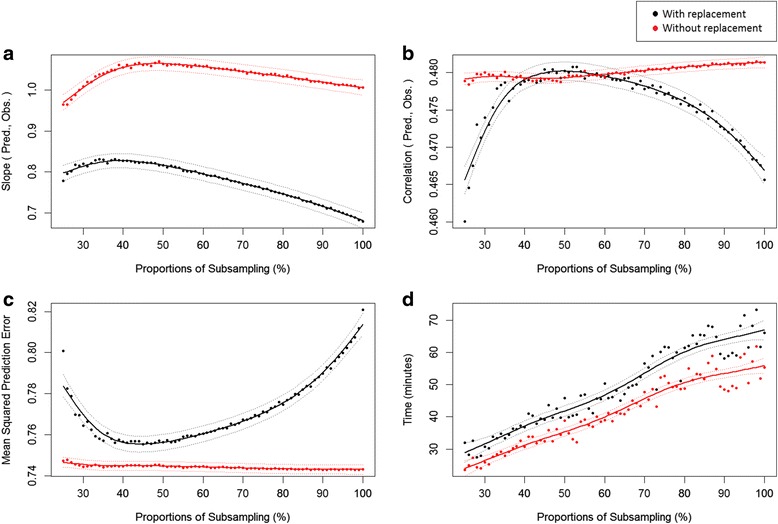
Prediction metrics (y axis) varying the amount of data under subsampling (x axis). Average across four datasets. **a** Bias as the slope between predicted and observed values; **b** Predictive ability as the correlation between predicted and observed values; **c** Mean squared prediction error; and **d** Computation time to fit the linear model

The mean outcome of prediction metrics across datasets is presented in Fig. 1. The results by individual dataset are presented in the Additional file 1. Numeric results for some proportions of subsampling are presented in Table 2.

**Table 2:** Summary of prediction metrics with for the complete dataset (Complete), and subsampling 50% with replacement (wR), and 33 and 50% without replacement (woR)

	Time (min.)	$\mathrm{C}\mathrm{o}{\mathrm{r}}_{\mathrm{y},\widehat{\mathrm{y}}}$	MSPE	${\upbeta}_{\mathrm{y},\widehat{\mathrm{y}}}$
Complete	55.90	0.4814	0.7431	1.0058
woR 33%	27.90	0.4794	0.7454	1.0239
woR 50%	35.32	0.4794	0.7447	1.0642
wR 50%	41.84	0.4802	0.7562	0.8161

$\mathrm{C}\mathrm{o}{\mathrm{r}}_{\mathrm{y},\widehat{\mathrm{y}}}$, correlation between observed and predicted value; MSPE, mean squared prediction error; ${\upbeta}_{\mathrm{y},\widehat{\mathrm{y}}}$, Prediction bias

### Computational improvement

The computational time had a linear response to subsampling (Fig. 1d). As expected, subsampling is clearly beneficial to speed up the computation of model fitting. The same trend was observed for individual datasets (Additional file 1). Although our evaluation of the improvement of computational performance used relatively small datasets, we believe the results must hold for larger datasets.

In comparison to fitting the model with whole data (Table 2), the computation time to fit the model at 50% subsampling was 33.6% faster with replacement and 58.3% faster without replacement. Yet, the computational cost was less than expected, once $3 p n\uppsi$ with $\uppsi =0.5$ should provide a model fitting 100% faster. This difference can be attributed to the computational cost of the sampling process along with the fixed cost of the initial problem settings. Computationtime 100% faster was achieved for subsampling 33% (or less) without replacement.

Interestingly, subsampling with replacement presented a slightly higher computational cost, also presenting worse predictive properties for subsampling lower than 40% or higher than 60%.

### Implications of subsampling on prediction parameters

#### Bias

The use of the complete dataset was nearly unbiased (Table 2). Subsampling with replacement was biased downwards, presenting the least bias at 40% replacement (${\upbeta}_{\mathrm{y},\widehat{\mathrm{y}}}=0.824$). Subsampling without replacement presented slight upward bias, being 1.8 and 5.8% more biased than the complete dataset at 33 and 50% subsampling, respectively.

#### Predictive ability

Across datasets (Table 2), the loss in predictive ability was negligible. Correlation between predictions and observations decreased 0.2% by subsampling with replacement at 50% subsampling, and 0.4% without replacement at both 33 and 50% subsampling.

#### MSPE

The negative impact on MSPE due to subsampling was also negligible. An increase of 0.3 and 0.2% were observed at 33 and 50% subsampling without replacement (Table 2). The impact of subsampling on MSPE was slightly higher with replacement, increasing 1.76% at 50% subsampling.

#### Dataset specific analysis

Although negligible, we observed a slight improvement in predictive ability and MSPE for all datasets at some optimal subsampling point. The optimal subsampling and respective improvement in predictive ability and MSPE are presented in Table 3.

**Table 3:** Optimal sampling observed for individual datasets to enhance predictive ability (PA) and mean squared prediction error (MSPE). Subsampling performed with (wR) and without replacement (woR)

	Optimal PA	Increase in PA	Optimal MSPE	Decrease in MSPE
Mouse	wR 66%	2.5%	woR 32%	<0.1%
Soybean	woR 25%	0.1%	woR 25%	0.1%
Wheat	woR 34%	0.7%	woR 33%	0.5%
Simulated F_2_	wR 87%	0.1%	wR 66%	0.2%

## Discussion

### Prediction machinery

Any algorithm that enhances prediction or computation performance is valuable for machine learning. At its optimal utilization, SBMC has the potential of improving prediction while reducing the computational cost [14]. However, reported results vary regarding any prediction improvement provided by subsampling [8, 24]. Subsampling has not been investigated in big data, for neither large $n$ nor large $p$, and that is a specific niche where subsampling may work best.

Previous studies indicate that there are no guarantees that SBMC will improve prediction, but it at least provides results equivalent to the whole dataset; however, we showed that subsampling can also provide a positive outcome for genomic prediction besides the computational aspects (Table 3), where the improvement reached 2.5% for the mouse data. We recommend including a bagging WGR with 50% subsampling without replacement in cross-validation studies looking for the most accurate prediction model.

### Random data

An interesting statistical property provided by SBMC is that data is sampled from a larger set, which is associated with that definition of a random term. This occurs because the observations used to update parameters are sampled from the empirical distribution of the data. This property violates the Bayesian assumption that data are *given*.

In classical Bayesian analysis, inferences are made based upon the posterior distribution of *parameters given data*, whereas random data implies that the parameters are sampled from the distribution of parameters given the current state of data. MCMC drives the posterior towards a relative entropy, possibly with larger sample variance associated with the continuous resampling used to update parameters with different subsets of data, but without obvious implications for the interpretation of the results [25].

### Incompleteness of data

Geyer [26] discussed the issue of subsampling Markov chains concluding “one does not get a better answer by throwing away data.” Nevertheless, he emphasizes that the value of the technique is 1) the reduction of dimensionality of $n$, and 2) the reduction of auto-correlation among chains.

Our counterargument is that the all data are used in the course of model fitting, although not simultaneously. In addition, accurate estimates are obtained when the subsampling strategy is used correctly [14]. We show that subsampling is a valid approach for genomic prediction purposes to fit high-dimensional models ($p\gg n$).

### Future directions

Subsampling uses only part of the data to fit the model in each MCMC round, that enables the computation of prediction statistics with the subset left out, which is referred to as out-of-bag statistics (OOB) [27]. The information provided by OOB is similar to the outcome of a cross-validation, with the advantage of being computed during the model fitting. Therefore, OOB could be used to re-weight observations (i.e., boosting). Another possibility is to adapt SBMC to other learning methods, such as elastic net [28], where OOB statistics could be utilized in the search for the tuning parameters without having to perform explicit cross-validation [29].

## Conclusion

SBMC decreases computation time without compromising prediction properties. We observed that subsampling approximately 33–50% without replacement and 40–60% with replacement in each round of MCMC is advantageous for fitting the model. Subsampling can dramatically reduce computational burden with little reduction in accuracy and, in some cases, enhanced predictive properties. This study provides insight into a general method for incorporating a particular type of bagging ensemble into the Gibbs sampling of whole-genome regressions.

## Additional file

Additional file 1: Results presented by individual dataset **Figure S1**. Time to fit the model (y axis) varying the subsampling method (x axis). **Figure S2**. Prediction ability (y axis) varying the subsampling method (x axis). Methods include Bayesian ridge regression (BRR) with regular sampler, and SBMC subsampling from 25 to 100%, with and without replacement. **Figure S3.** Mean squared prediction error (y axis) varying the subsampling method (x axis). Methods include Bayesian ridge regression (BRR) with regular sampler, and SBMC subsampling from 25 to 100%, with and without replacement. **Figure S4.** Bias (y axis) varying the subsampling method (x axis). Methods include Bayesian ridge regression (BRR) with regular sampler, and SBMC subsampling from 25 to 100%, with and without replacement. (DOCX 232 kb)

## Abbreviations

CIMMYT: International Maize and Wheat Improvement Center
GBLUP: Genomic best linear unbiased prediction
GSRU: Gauss-Seidel residual updating
GWP: Genome-wide prediction
LHS: Left-hand side
MCMC: Markov chain Monte Carlo
MSPE: Mean square prediction error
OOB: Out-of-bag statistics
REML: Restricted maximum likelihood
RHS: Right-hand side
SBMC: Subsampling bootstrap Markov chain
SNP: Single nucleotide polymorphism
