# Erratum to: Genomic prediction using subsampling

**DOI:** 10.1186/s12859-017-1629-5

**Published:** 2017-04-26

**Authors:** Alencar Xavier, Shizhong Xu, William Muir, Katy Martin Rainey

**Affiliations:** 1 Department of Agronomy, Purdue University, 915 W. State St., Lilly Hall, West Lafayette, IN 47907 USA; 2 Department of Plant Science, University of California, 3134 Batchelor Hall, Riverside, CA 92521 USA; 3 Department of Animal Science, Purdue University, 915W. State St., Lilly Hall, West Lafayette, IN 47907 USA

## Erratum

Following publication of this article [1], it has come to our attention that some of the equations included were distorted by a formatting issue in the PDF form. This issue affected Eqs. 8, 9 and 11. The correct forms of the equations are shown below.

8 $$ {\sigma}_{\mathrm{b}}^2\left|*\sim \frac{{\mathbf{b}}^{\hbox{'}}\mathbf{b} + {\mathrm{S}}_{\mathrm{b}}{\nu}_{\mathrm{b}}}{\chi_{\mathrm{p}+{\nu}_{\mathrm{b}}}^2}\kern0.24em \mathrm{and}\kern0.24em {\sigma}_{\mathrm{e}}^2\right|*\sim \frac{{\mathbf{e}}^{\hbox{'}}\mathbf{e} + {\mathrm{S}}_{\mathrm{e}}{\nu}_{\mathrm{e}}}{\chi_{\mathrm{n}+{\nu}_{\mathrm{e}}}^2} $$

9 $$ {\mathrm{b}}_{\mathrm{j}}^{\mathrm{t}+1}\Big|*\sim \mathrm{N}\left(\frac{{\overset{\sim }{\mathbf{x}}}_{\mathbf{j}}\hbox{'}{\overset{\sim }{\mathbf{e}}}^{\mathrm{t}}+\psi {\mathbf{x}}_{\mathrm{j}}\hbox{'}{\mathbf{x}}_{\mathrm{j}}{\mathrm{b}}_{\mathrm{j}}^{\mathrm{t}}\ }{\psi {\mathbf{x}}_{\mathrm{j}}\hbox{'}{\mathbf{x}}_{\mathrm{j}}+{\uplambda}_{\mathrm{j}}},\frac{\sigma_{\mathrm{e}}^2}{\psi {\mathbf{x}}_{\mathrm{j}}\hbox{'}{\mathbf{x}}_{\mathrm{j}}+{\uplambda}_{\mathrm{j}}}\right) $$

11 $$ {\sigma}_{\mathrm{e}}^2\Big|*\sim \frac{{\overset{\sim }{\mathbf{e}}}^{\hbox{'}}\overset{\sim }{\mathbf{e}} + {\mathrm{S}}_{\mathrm{e}}{\nu}_{\mathrm{e}}}{\chi_{\uppsi \mathrm{n}+{\nu}_{\mathrm{e}}}^2} $$
