# Supplementary material for: Genomic prediction using subsampling
Source: BMC Bioinformatics. 2017 Mar 24;18:191. doi: 10.1186/s12859-017-1582-3 (PMC5366167; doi:10.1186/s12859-017-1582-3)
Supplement: Additional file 1: — Results presented by individual dataset Figure S1. Time to fit the model (y axis) varying the subsampling method (x axis). Figure S2. Prediction ability (y axis) varying the subsampling method (x axis). Methods include Bayesian ridge regression (BRR) with regular sampler, and SBMC subsampling from 25 to 100%, with and without replacement. Figure S3. Mean squared prediction error (y axis) varying the subsampling method (x axis). Methods include Bayesian ridge regression (BRR) with regular sampler, and SBMC subsampling from 25 to 100%, with and without replacement. Figure S4. Bias (y axis) varying the subsampling method (x axis). Methods include Bayesian ridge regression (BRR) with regular sampler, and SBMC subsampling from 25 to 100%, with and without replacement. (DOCX 232 kb) [file 12859_2017_1582_MOESM1_ESM.docx]

**Supplementary File S1:** Results presented by individual dataset


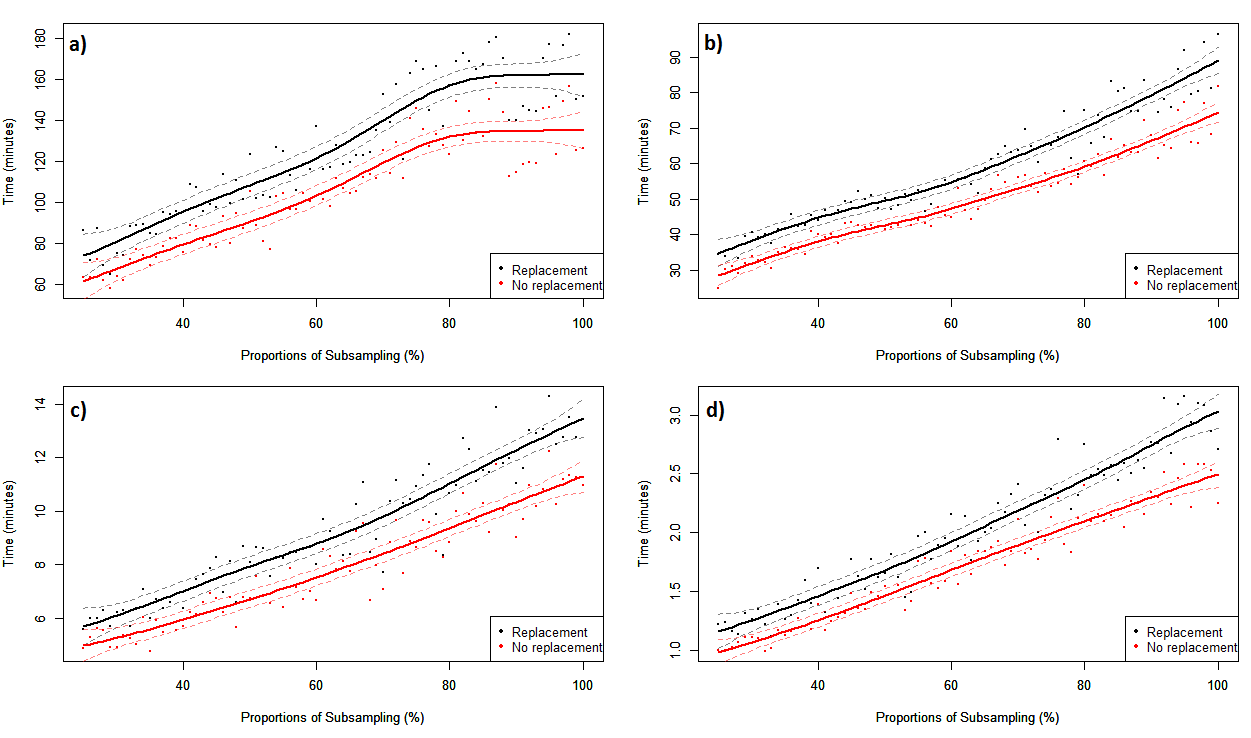


**Figure 1.** Time to fit the model (y axis) varying the subsampling method (x axis). Methods include Bayesian ridge regression (BRR) with regular sampler, and SBMC subsampling from 25% to 100%, with and without replacement. Datasets correspond to **a)** mouse; **b)** soybean; **c)** wheat; and **d)** simulated F2.


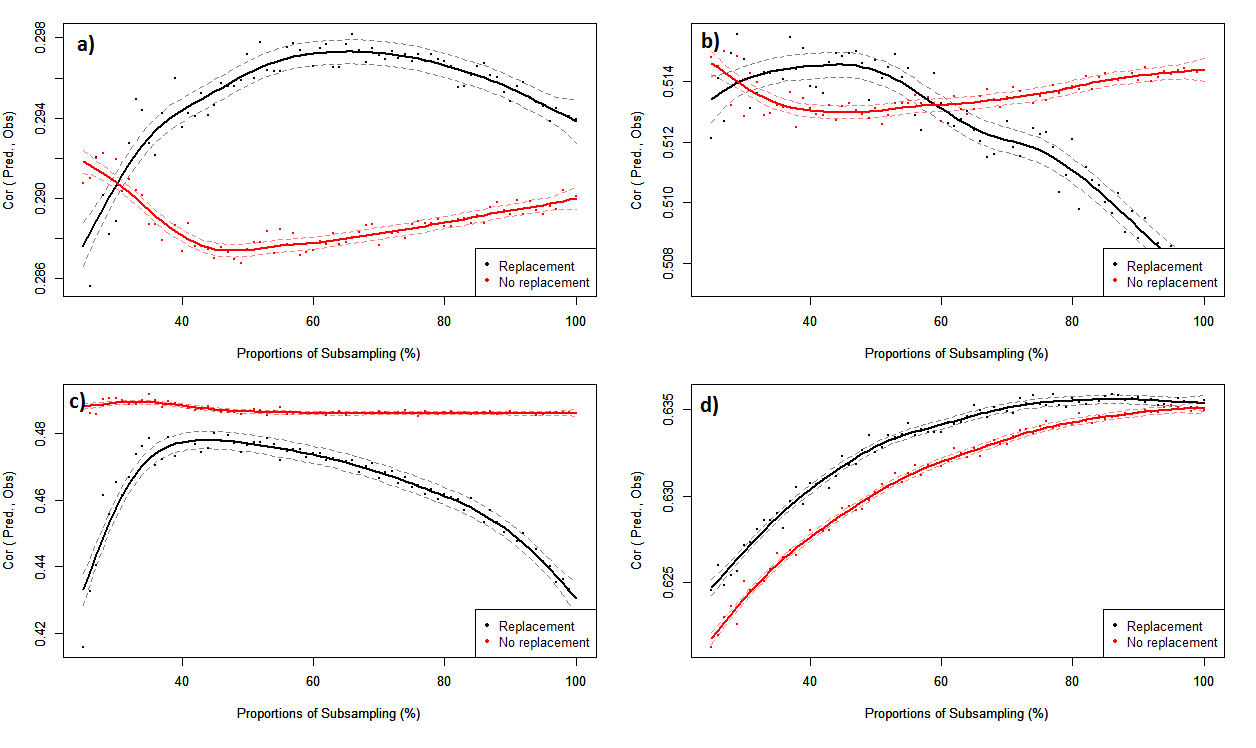


**Figure 2.** Prediction ability (y axis) varying the subsampling method (x axis). Methods include Bayesian ridge regression (BRR) with regular sampler, and SBMC subsampling from 25% to 100%, with and without replacement. Datasets correspond to **a)** mouse; **b)** soybean; **c)** wheat; and **d)** simulated F2.


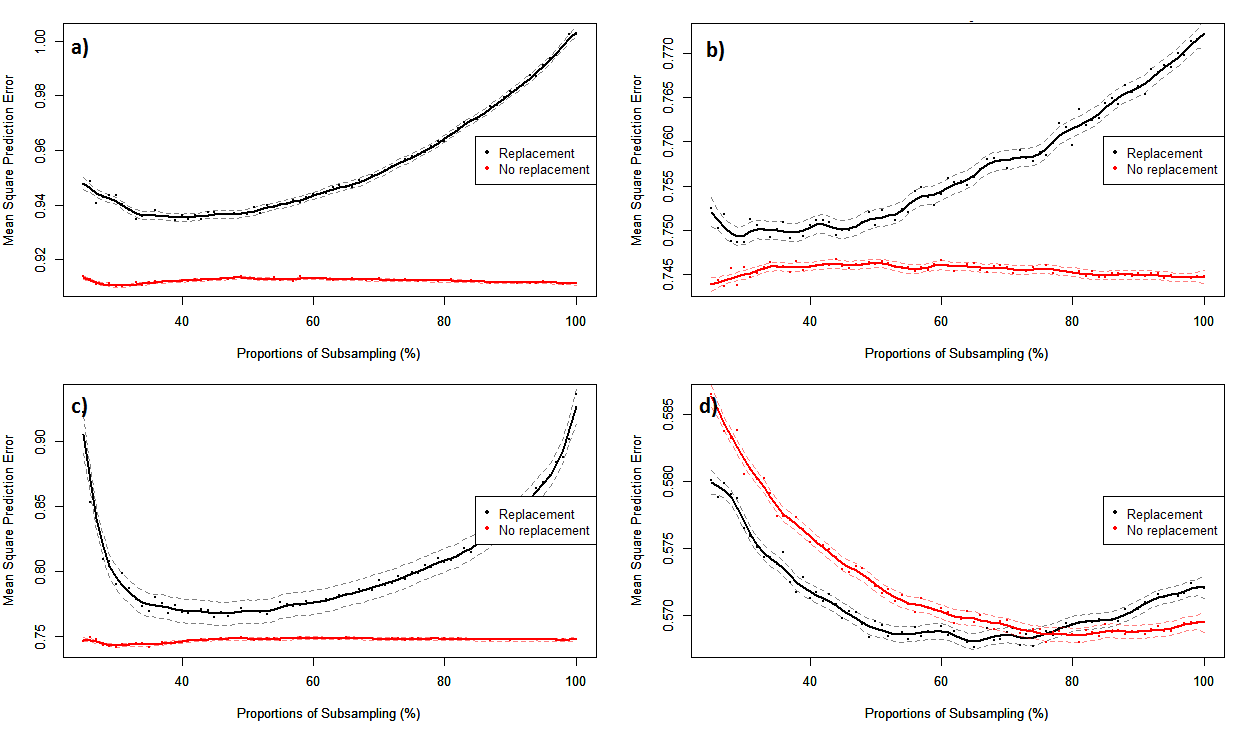


**Figure 3.** Mean squared prediction error (y axis) varying the subsampling method (x axis). Methods include Bayesian ridge regression (BRR) with regular sampler, and SBMC subsampling from 25% to 100%, with and without replacement. Datasets correspond to **a)** mouse; **b)** soybean; **c)** wheat; and **d)** simulated F2.


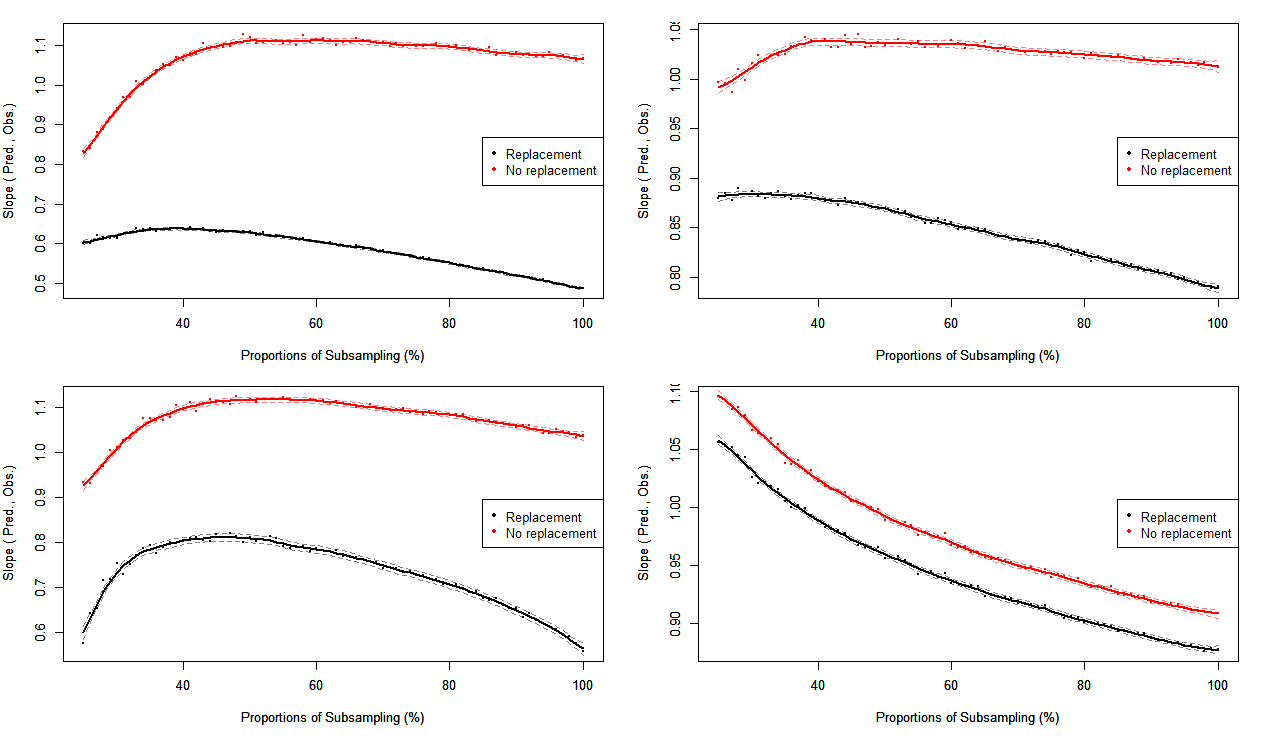


**Figure 4.** Bias (y axis) varying the subsampling method (x axis). Methods include Bayesian ridge regression (BRR) with regular sampler, and SBMC subsampling from 25% to 100%, with and without replacement. Datasets correspond to **a)** mouse; **b)** soybean; **c)** wheat; and **d)** simulated F2.
